# Multifilament spinning of mechanically recycled polypropylene from post-consumer sources for a circular economy in textile applications

**DOI:** 10.1038/s41598-025-98375-4

**Published:** 2025-04-23

**Authors:** Leopold Alexander Frankenbach, Stephanie Lukoschek, Iris Kruppke, Chokri Cherif

**Affiliations:** 1 Institute of Textile Machinery and High Performance Material Technology (ITM), Technical University Dresden, 01062 Dresden, Germany; 2 Centre for Tactile Internet with Human-in-the-Loop (CeTI), TUD Dresden University of Technology, 01069 Dresden, Germany

**Keywords:** Multifilament-spinning, Recycling, Sustainable textiles, Polypropylene, Yarns, Post-consumer, Mechanical engineering, Environmental impact

## Abstract

The use of polypropylene (PP) waste in the textile industry has been limited to low-value applications, as it is difficult to produce high-quality recycled fibers. Recent advances in recycling and purification technologies have enabled the production of recycled polypropylene (rPP) suitable for textile-grade fibers. This study focuses on the melt spinning of rPP multifilament yarns obtained from post-consumer waste and evaluates the influence of critical spinning parameters such as godet temperature, quenching conditions, draw ratio and draw down speed on yarn properties. The test results show that highly purified rPP can be successfully spun into multifilament yarns with a tensile strength of up to 4.2 cN/dtex and an elongation at break of 20%. These values demonstrate the potential of using post-consumer PP in high-performance applications, overcoming the traditional limitations associated with recycled PP. This work provides a pathway for expanding the use of post-consumer waste in the production of high value textile fibers and promotes a more sustainable approach to polypropylene waste management.

## Introduction

The ever-increasing amount of waste produced globally, largely driven by urbanization and changing consumer lifestyles, poses a significant environmental challenge. A substantial portion of this waste consists of plastic, which is predominantly disposed of in landfills or released into the marine environment, resulting in widespread environmental pollution and the persistence of small plastic particles in ecosystems. Over the past two decades, global plastic waste recycling has shown notable growth, driven primarily by organisation for economic co-operation and development-countries (OECD) in the European Union, as well as by India and China. By 2019, these regions had achieved recycling rates of 12–13%^1^. In contrast, non-OECD Asian countries and Latin America have experienced slower progress. The United States and the Middle East & North Africa region have made minimal advancements, with the US reaching only a 4.5% recycling rate by 2019, according to OECD data^1^. Promoting the recycling of polypropylene (PP) and other polymeric materials is crucial to mitigate the impact on the environment and reduce the depletion of natural resources. Given the significant volume of plastic waste generated annually, it is critical to differentiate between the two primary sources: post-industrial and post-consumer plastic waste. Post-industrial plastic waste primarily originates from industrial processes, such as production scrap or off-cuts from manufacturing, and is typically of higher and more consistent quality. In contrast, post-consumer plastic waste is derived from discarded consumer products, such as packaging materials, electronic devices, and household items. Due to its varied composition, exposure to environmental conditions, and the presence of contaminants like food residues or mixed polymer types, post-consumer waste presents greater challenges for recycling into high-quality materials. Nonetheless, post-consumer plastic waste represents the largest proportion of plastic waste globally^2^, making its effective utilization crucial for achieving sustainability goals^3^ and advancing a circular economy. Extensive research and literature have been dedicated to the three primary plastic recycling technologies: mechanical recycling^4–6^, chemical recycling^7–9^ and energy recycling^10–12^. Recycling and reusing plastic waste are more sustainable approaches than landfilling or incineration, but they are hindered by the presence of various contaminants, especially in the packaging industry. Pre-treatment techniques like purification by washing^13^, dehalogenation^14^ or plastic waste separation technologies^15,16^ are emerging as viable solutions to improve the quality of recycled plastics. Some innovative recycling applications include using plastic waste in the construction industry for materials such as paving blocks^17^, ceramics^18^, and as feedstock for various industrial processes. To address the growing volume of plastic waste, it is essential to consider not only the industrial applications but also the diverse sources of post-consumer plastic waste generated by households and everyday consumer activities. Post-consumer plastic waste encompasses a broad spectrum of products, ranging from single-use packaging to durable household items, which require tailored recycling solutions to manage effectively. Due to the inherently short lifespan of many plastic products, considerable quantities of such waste are generated every year. The average lifespan of plastic products is approximately ten years, though this varies greatly by the application. While plastic applications used in the industrial machinery and construction sectors can remain in use for decades, those used for packaging are typically in use for less than one year^19^. Plastic packaging applications are often used just once before they are disposed of, which is one of the reasons why plastic packaging is the main source of plastic waste worldwide. Despite the considerable volume of post-consumer food packaging waste, effective recycling strategies for these materials remain largely undeveloped^20^. Currently, there is no established approach within the textile industry to repurpose post-consumer food packaging into textile products. However, recent initiatives indicate the potential for progress. For instance, some studies have shown that composite materials, incorporating post-consumer polypropylene and cotton, can be processed into staple fibers^21^. By refining the melt spinning process, companies like *IGF Asota*^22^ have been able to produce melt spun PP yarns with a post-industrial PP content, demonstrating the feasibility of integrating recycled polymers into high-quality fibers. Studies have explored various reinforcement methods and compatibilization strategies to address the inherent limitations of recycled polymers. For example, incorporating short hemp fibers (SHFs) into rPP has demonstrated enhanced stiffness and improved fiber-matrix interaction when compatibilizing agents like maleic anhydride grafted polypropylene (PP-g-MA) are used^23^. Furthermore, recent work on rPP fibers for concrete reinforcement demonstrated that optimized processing conditions can yield rPP fibers with sufficient strength and durability for structural applications, providing an environmentally friendly alternative to virgin polypropylene​^24,25^. Other studies have focused on integrating natural fibers such as bamboo into rPP matrices, which revealed that chemical treatment of bamboo fibers can significantly improve the composite’s compatibility and mechanical performance^26^. Recent studies have explored the potential of recycled polypropylene in various manufacturing processes. For instance, Fused Deposition Modelling (FDM) has been used to process rPP/aluminum powder composites, demonstrating improved mechanical properties and sustainability^27^. The utilisation of post-consumer plastics in high-performance applications is constrained by their heterogeneous composition, contamination, and degradation, thus rendering post-industrial waste the preferred alternative. Previous studies have demonstrated that the addition of fibres or compatibilisers can enhance rPP composites; however, research on directly melt-spinning post-consumer rPP into high-performance yarns remains limited. The present study endeavors to develop optimized processing techniques to enhance the quality and mechanical performance of post-consumer recycled rPP for textile applications. By systematically analyzing the influence of key melt-spinning parameters—such as draw-down speed, draw ratio, and drawing temperature—on the mechanical properties of rPP multifilament yarns, the research provides a comprehensive framework for improving yarn quality. The optimization of these critical parameters is expected to ensure the competitiveness of rPP yarns with virgin polymer-based alternatives and facilitate their adoption in high-value textile sectors. This approach is expected to support the broader industrial use of rPP and contribute to the development of a more sustainable and circular economy.

## Experimental section

### Materials

For this study, two distinct types (Systalen^®^ 32.9002 and Systalen^®^ 32.0999) of recycled polypropylene (rPP) were sourced from post-consumer packaging waste, provided by Der Grüne Punkt Holding GmbH & Co. KG, Köln/Germany. These rPP materials were derived from the “Gelber Sack” collection system in Germany, which is designed for the sorting and recycling of plastic waste from households. The selected rPP types have been processed using a specialized treatment method that integrates advanced technologies to enhance the quality of the recycled material. This process involves precise color sorting of plastic flakes to improve aesthetic consistency, optimized washing procedures to ensure the removal of impurities, and an advanced odor elimination system to reduce any residual odors commonly associated with recycled plastics. These enhanced methods aim to improve the performance and applicability of rPP for high-quality applications, making them suitable for further investigation in polymer processing and melt spinning.

### Measuring, test and machine equipment

Thermal analyses were performed using thermogravimetric analysis (TGA, Q500, TA Instruments, New Castle, DE, USA) and differential scanning calorimetry (DSC, Q2000, TA Instruments, New Castle, DE, USA). The thermal decomposition of the materials prior to melt spinning was evaluated in the temperature range of 30 °C to 800 °C under an air atmosphere, with a heating rate of 40 K/min. In addition, the heat flow of all materials was measured from 30 °C to 400 °C under a nitrogen atmosphere, employing a modulated heating rate of 5 K/min. The use of the second heating cycle in DSC ensured that the material was fully relaxed, eliminating any prior thermal history, such as residual stress or crystallization behavior from previous processing. The rheological properties were assessed with an extrusion plastometer (MFLOW, ZwickRoell, Ulm, Germany) in accordance with ISO-1133. Mechanical properties of the multifilaments were determined by tensile testing on a Zwick2.5 testing machine (ZwickRoell, Ulm, Germany), using a gauge length of 125 mm and a testing speed of 125 mm/min. The rPP multifilament yarns were spun on a modular melt spinning system from Dienes Apparatebau GmbH, Mühlheim am Main, Germany. This system consists of a ZSE-MAXX18 twin-screw extruder (Leistritz Extrusionstechnik, Nuremberg, Germany), two melt pumps (Maag Germany GmbH, Grossostheim, Deutschland) with a pump capacity of 2.642 cm³ and a spinning package (40 filaments, L/D = 3, d = 0.3 mm) manufactured by Sossna GmbH, Dorsten, Germany. The yarn runs over 5 godet draw duos (DD) and is wound with a Sahm 3200 (Georg Sahm GmbH & Co. KG, Eschwege, Germany) winder.

### Process parameters

#### Process parameters for the extrusion level

**Fig. 1:**
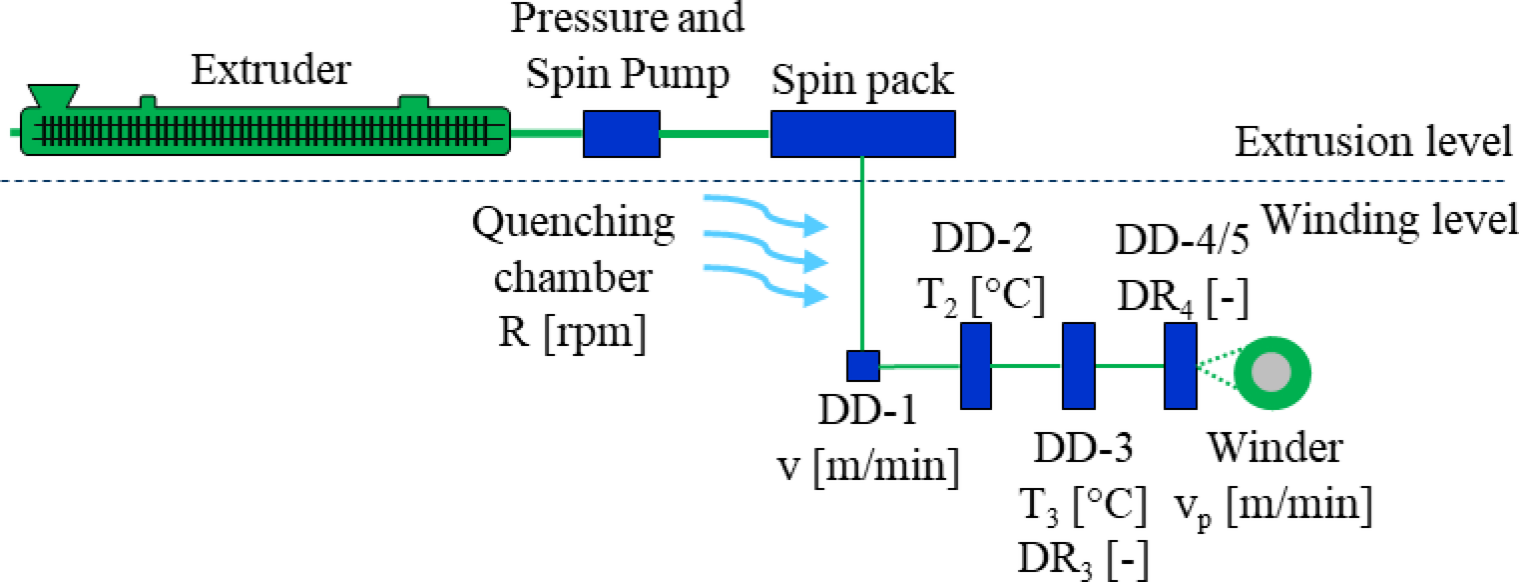
The following schematic provides a comprehensive overview of the melt spinning system. The system is divided into extrusion and winding levels. The speed of the quenching chamber (R), draw-off speed (v), the temperature (T_2_) on the second DD, the temperature (T_3_) and draw ratio (DR_3_) on the third DD, the draw ratio (DR_4_) on the fourth DD, and the process speed (v_p_) are all indicated.

During the extrusion level (Fig. 1), process parameters were carefully controlled to ensure consistency across all trials. The extrusion and winding level was equipped with state-of-the-art process control technology, ensuring precise regulation of temperature, pressure, and material flow. Real-time monitoring facilitated immediate adjustments, thereby minimizing fluctuations and ensuring the maintenance of optimal processing conditions. This high level of automation and control guaranteed reproducibility across all trials and ensured consistently high filament quality. The extruder operated at a constant screw speed of 100 revolutions per minute (rpm), which facilitated the continuous flow of the polymer melt. The spinning pump, responsible for delivering the polymer melt to the spinneret, maintained a consistent speed of 20.7 rpm, ensuring a stable and precise feed rate. The melt temperature was set at 250 °C. The spin pack temperature was also maintained at 250 °C to ensure uniform temperature distribution across the polymer stream before extrusion through the spinneret. The spinning pressure was kept constant at 100 bar. This pressure facilitated the smooth extrusion of the molten polymer, contributing to the formation of consistent, high-quality filaments during the melt spinning process. The process parameters at the winding level were adapted to the five specific trials.

The selection of process parameters was guided by prior literature on PP melt spinning^28,29^, which provided baseline values for key variables such as draw-down speed, draw ratio, and thermal conditions. These parameters were further refined through controlled experimental trials to ensure optimal fiber quality and reproducibility.

#### Process parameters for draw down speed-trials

The primary objective of the first series of tests was to examine the effect of varying draw-down ratios on the mechanical properties of the produced multifilament yarns. Draw-down speed v is a critical parameter in melt spinning, as it directly influences the degree of molecular orientation and crystallinity of the filaments, thereby affecting their tensile strength, elongation, and overall performance. According to polymer deformation theory, increasing the draw-down speed induces higher extensional flow, which enhances polymer chain alignment along the fiber axis, leading to improved mechanical properties^30,31^. By systematically adjusting the draw-down speed, the study aimed to identify optimal conditions for achieving the desired balance between strength and flexibility in the yarns. The draw ratio is a key factor in determining the final molecular arrangement, as higher draw ratios typically result in greater crystalline phase development while reducing amorphous content. This transition from an amorphous to a more ordered structure is essential for increasing yarn tenacity but often comes at the cost of reduced elongation. Table 1 outlines the key process parameters employed at the winding stage, which included variations in the take-up speed and the tension applied to the yarns during winding. These parameters were carefully controlled to ensure consistency across trials, allowing for an accurate comparison of the mechanical properties at different draw-down ratios.

**Table 1:** Process parameters for draw down speed-trials.

Trial-ID	*R* [rpm]	v [m/min]	T_2_ [°C]	T_3_ [°C]	DR_3_ [-]	DR_4_ [-]	v_*P*_ [m/min]
32.0999-1	500	750	30	30	1	1	750
32.0999-2	500	1000	30	30	1	1	1000
32.0999-3	500	1250	30	30	1	1	1250
32.0999-4	500	1500	30	30	1	1	1500
32.0999-5	500	1750	30	30	1	1	1750
32.0999-6	500	2000	30	30	1	1	2000
32.9002-1	500	750	30	30	1	1	750
32.9002-2	500	1000	30	30	1	1	1000
32.9002-3	500	1500	30	30	1	1	1500
32.9002-4	500	2000	30	30	1	1	2000
32.9002-5	500	2500	30	30	1	1	2500

#### Process parameters for draw ratio-trials

The aim of the second series of trials was to explore the impact of various draw ratios on the mechanical properties of the spun multifilament yarns. The draw ratio, defined as the ratio of the final filament length to its initial length, is a critical parameter that governs the degree of polymer chain orientation and crystallinity within the filaments. This, in turn, directly influences the yarn’s mechanical performance, including tensile strength, modulus, and elongation at break. According to the fundamentals of fiber formation, increasing the draw ratio enhances molecular alignment along the fiber axis, reducing chain entanglements and promoting crystallization^32^. By systematically varying the draw ratios in this test series, the study aimed to optimize the stretching process during spinning, ensuring enhanced filament orientation, crystallinity, and structural integrity. The following Table 2 presents the process parameters set during the winding stage, which included variations in draw ratios and winding speed. These controlled variables ensured that the impact of each draw ratio could be systematically assessed, providing a comprehensive understanding of its influence on yarn mechanical performance.

**Table 2:** Process parameters for draw ratio-trials.

Trial-ID	*R* [rpm]	v [m/min]	T_2_ [°C]	T_3_ [°C]	DR_3_ [-]	DR_4_ [-]	v_*P*_ [m/min]
32.0999-7	500	750	60	130	1.1	1.818	1500
32.0999-8	500	750	60	130	1.1	2.424	2000
32.0999-9	500	750	60	130	1.1	3.030	2500
32.0999-10	500	750	60	130	1.1	3.636	3000

#### Process parameters for draw temperature-trials

The third series of experiments aimed to investigate the effect of the order of the drawing ratios on the mechanical properties of the spun multifilament yarns. The godet system, which controls the drawing process by regulating filament tension and stretching ratios, plays a crucial role in defining fiber morphology and mechanical characteristics. The draw ratio applied at different stages influences the degree of polymer chain orientation and crystallinity within the fibers. A single-stage drawing process imposes a higher strain in a shorter timeframe, potentially leading to structural irregularities and reduced molecular alignment. Conversely, a two-stage drawing process allows for a more gradual realignment of polymer chains, leading to improved stress distribution and higher crystalline phase formation. The following Table 3 presents the process parameters used during the winding stage, detailing the drawing sequences, winding speeds, and applied tensions.

**Table 3:** Process parameters for draw temperature-trials.

Trial-ID	*R* [rpm]	v [m/min]	T_2_ [°C]	T_3_ [°C]	DR_3_ [−]	DR_4_ [−]	v_*P*_ [m/min]
32.0999-11	500	750	60	60	3.03	1.1	2500
32.0999-12	500	750	60	90	3.03	1.1	2500
32.0999-13	500	750	60	130	3.03	1.1	2500

#### Process parameters for godet-trials

The fourth series of tests was designed to investigate the effect of the order of the drawing ratios on the mechanical properties of the spun multifilament yarns. The following table presents the process parameters used during the winding stage, detailing the drawing sequences, winding speeds, and applied tensions. These parameters (Table 4) were carefully adjusted to ensure that the influence of the drawing sequence on the mechanical properties of the yarns could be systematically evaluated, providing valuable insights into the optimization of the melt spinning process.

**Table 4:** Process parameters for godet-trials.

Trial-ID	*R* [rpm]	v [m/min]	T_2_ [°C]	T_3_ [°C]	DR_3_ [−]	DR_4_ [–]	v_*P*_ [m/min]
32.0999-14	500	750	60	130	1.100	1.818	1500
32.0999-15	500	750	60	130	1.818	1.100	1500
32.0999-16	500	750	60	130	2.000	1.000	1500
32.0999-17	500	750	60	130	2.666	1.000	2000
32.0999-18	500	750	60	130	3.000	1.000	2250
32.0999-19	500	750	60	130	3.333	1.000	2500

#### Process parameters for quenching -trials

The aim of the fifth series of tests was to investigate the influence of cooling air flow (quenching air) on the mechanical properties of the spun multifilament yarns. Quenching air plays a critical role in controlling the solidification rate of extruded filaments, influencing their morphological structure, crystallization behavior, and final mechanical performance^28^. During melt spinning, rapid cooling typically results in lower crystallinity and increased amorphous content, leading to fibers with higher elasticity but reduced tensile strength. Conversely, slower cooling allows for more ordered crystalline phase formation, which enhances mechanical strength but may reduce ductility. The following Table 5 presents the process parameters of the winding level, detailing the variations in quenching air conditions and their potential influence on fiber structure.

**Table 5:** Process parameters for quenching-trials.

Trial-ID	*R* [rpm]	v [m/min]	T_2_ [°C]	T_3_ [°C]	DR_3_ [−]	DR_4_ [−]	v_*P*_ [m/min]
32.9002-6	500	1500	30	30	1	1	1500
32.9002-7	1000	1500	30	30	1	1	1500
32.9002-8	1500	1500	30	30	1	1	1500

## Results and discussion

### Thermal and rheological properties

**Fig. 2:**
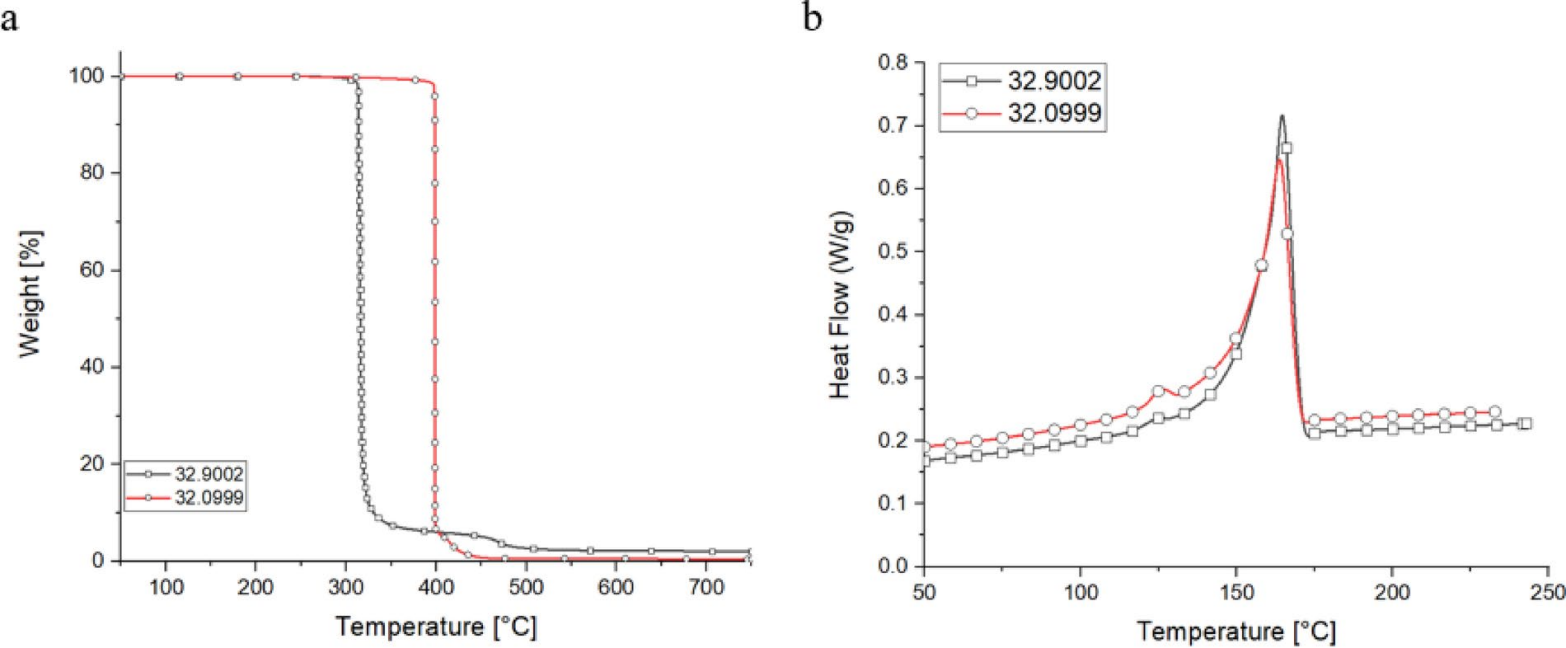
TGA (**a**) and DSC (**b**) of the rPP materials.

The two materials that were to be melt-spun were characterized (Fig. 2) using TGA and DSC.

**Fig. 3:**
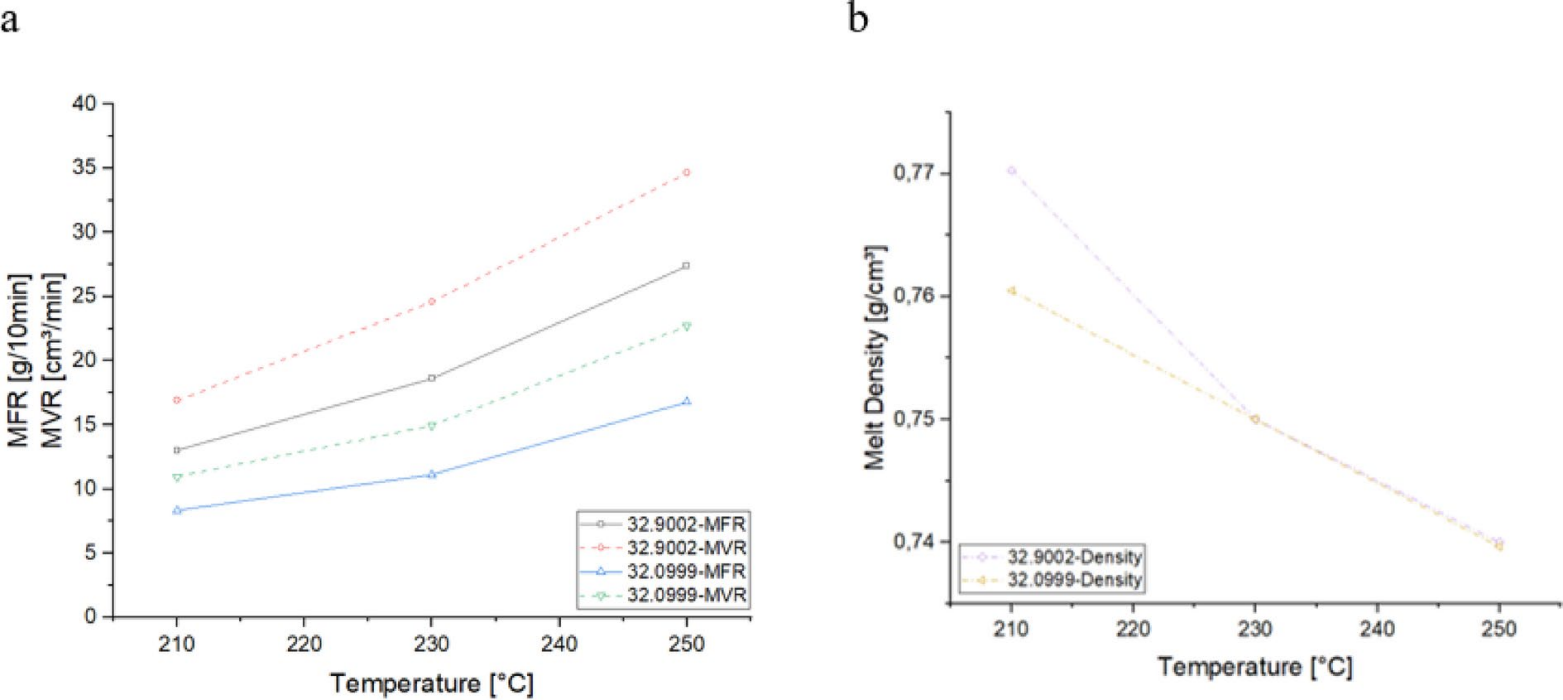
The material properties of rPP, including the melt flow rate (MFR) and the melt volume ratio (MVR) (**a**), as well as the melt density (**b**).

No decomposition is observed in the TGA curves for either material up to approximately 300 °C, indicating that both materials remain thermally stable up to this point. For material 32.9002, a significant degradation phase begins at around 320 °C, where the material undergoes rapid mass loss, dropping from 100% to approximately 10% in weight. Following this major degradation, smaller, less pronounced degradation stages continue up to 500 °C, suggesting the breakdown of residual components or further thermal degradation of byproducts. In contrast, material 32.0999 exhibits a large degradation phase starting around 400 °C, extending to about 450 °C, where a substantial portion of the material degrades. These thermal behaviors highlight the importance of maintaining the processing temperature below 300 °C to avoid premature thermal decomposition. The clear onset of degradation at temperatures above 300 °C for both materials indicates that processing at higher temperatures could result in the degradation of polymer chains, leading to a loss of mechanical properties and compromising the quality of the final product. Therefore, to ensure the integrity of the materials during melt spinning, the processing temperature should be carefully controlled to remain well below this threshold. Both materials exhibited a well-defined melting range between 150 and 175 °C, as indicated by the DSC curves. This pronounced melting range suggests that the polymer chains undergo significant thermal transitions within this temperature window, making it a critical phase for material processing. The onset of melting at 150 °C indicates the beginning of polymer chain mobility, while the complete melting is achieved by 175 °C, at which point the materials have transitioned into a fully molten state. Given these thermal characteristics, the processing window for both materials can be defined as between 175 and 300 °C. Therefore, careful control of processing temperatures within this window is crucial for maintaining material integrity and ensuring high-quality filament production. The following Fig. 3 illustrates the Melt Flow Index (MFI) values for the two materials, measured in accordance with the ISO 1133 standard for polypropylene at two different temperatures, 210 °C and 250 °C. The MFI, which includes both the Melt Flow Rate (MFR) and Melt Volume Rate (MVR), is a key indicator of the polymer’s flowability under specified conditions. While the MFR represents the mass of polymer extruded per unit time, the MVR describes the corresponding volume flow, making both parameters essential for assessing the processability of the material during melt spinning.

A clear temperature-dependent increase in flowability is observed for both materials. Material 32.9002, with an MFR_210°C, 2.16 kg_ of 13 g/10 min, exhibits a higher flowability compared to material 32.0999, which has an MFR_210°C, 2.16 kg_ of 8 g/10 min at the same conditions. This difference indicates that material 32.9002 is less viscous and flows more easily than 32.0999 under the same processing conditions. For both materials, the flowability increases by a factor of approximately 1.4 for every 10 K rise in temperature. This trend highlights the significant influence of temperature on the melt behavior of these polymers, with higher temperatures resulting in reduced viscosity and enhanced flow characteristics. Additionally, the density of both materials shows a temperature dependency, decreasing by roughly 3% for every 10 K increase in temperature. This reduction in density with increasing temperature is typical for polymers as they expand and their molecular structure becomes more mobile. These findings underscore the importance of precise temperature control during processing to ensure optimal flow behavior and to achieve the desired material properties in applications such as melt spinning or molding.

### Visual properties

**Fig. 4:**
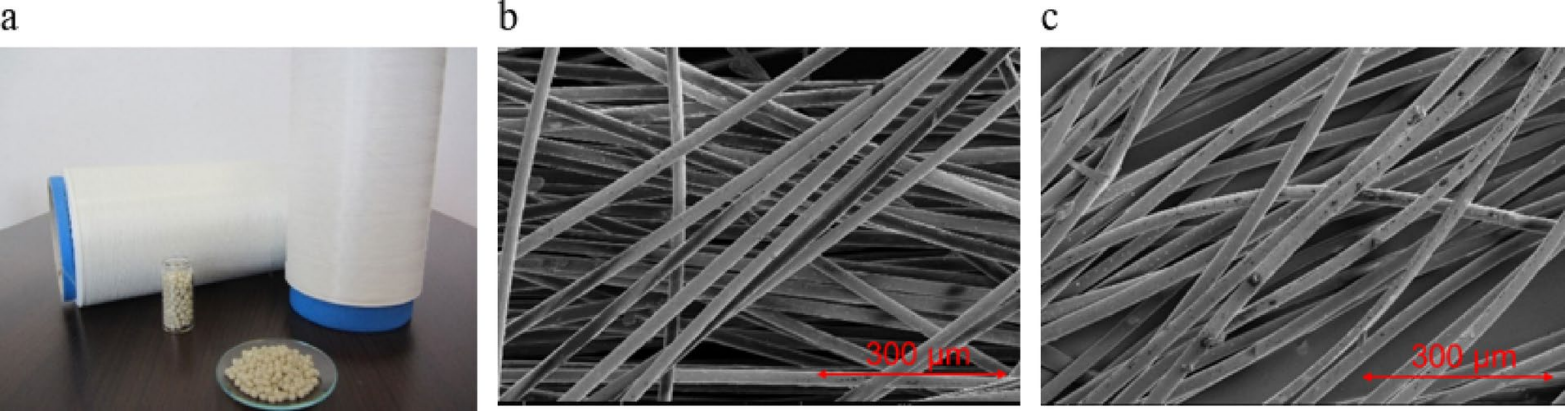
Yarns from post-consumer PP (**a**) and SEM-Image of the yarns from 32.0999 (**b**) and 32.9002 (**c**).

**Fig. 5:**
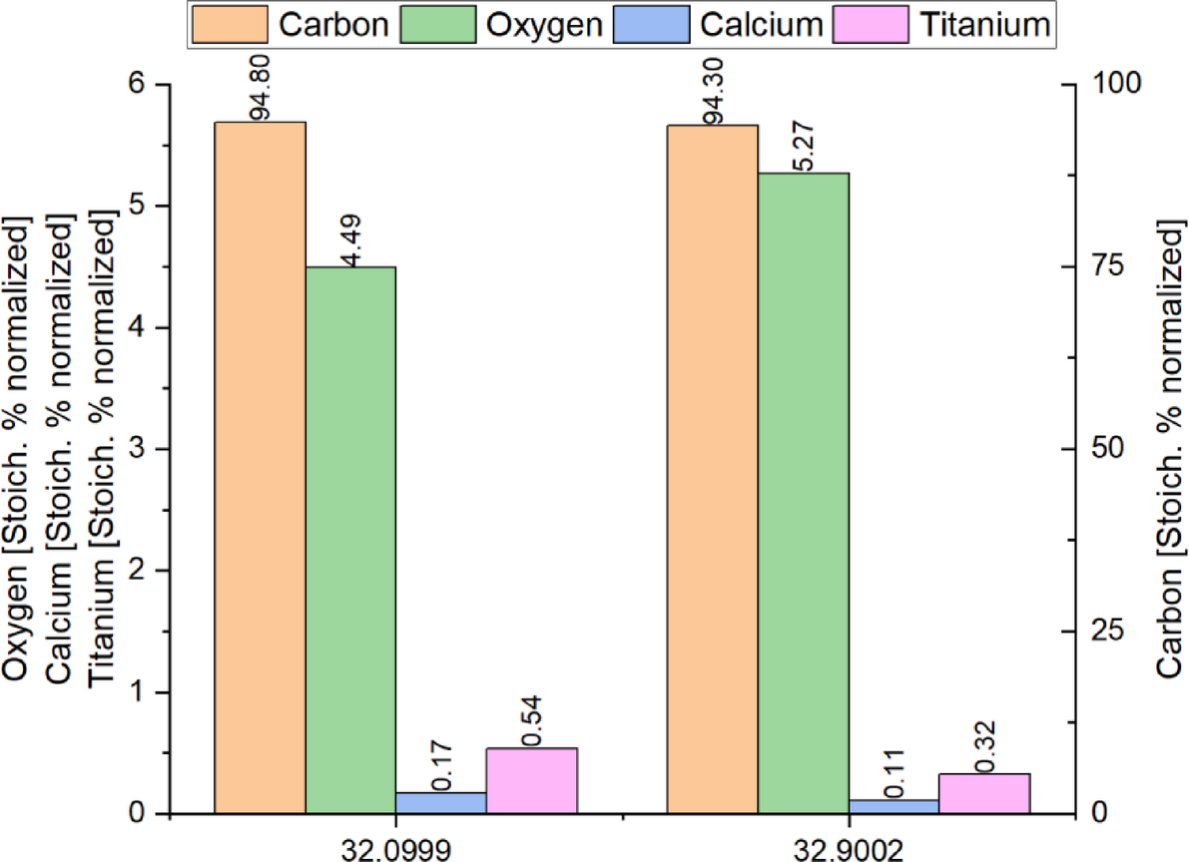
Stoichiometrically normalized elemental composition of samples 32.0999 and 32.9002, determined via EDX analysis.

Scanning Electron Microscopy (SEM) images (Fig. 4) revealed surface irregularities such as small spots and imperfections, which are characteristic of recycled polymer-based yarns due to repeated thermal and mechanical stress during the recycling process. The yarns made from material 32.9002 exhibit numerous small, bright particles on the surface. Based on energy-dispersive X-ray spectroscopy (EDX, Fig. 5), these particles contain titanium and calcium, suggesting the presence of inorganic additives or fillers such as titanium dioxide (TiO₂) and calcium carbonate (CaCO_3_), which were not fully dispersed or originate from the original post-consumer input materials. The distribution of these particles across the yarn surface may influence both mechanical and optical properties of the resulting filaments. In contrast, yarns made from material 32.0999 show a smoother surface with fewer visible particulate inclusions. This indicates a higher degree of homogeneity and material purity, likely due to more efficient contaminant removal and better filler dispersion during the recycling process.

### Mechanical properties

#### Mechanical properties of the yarns spun within the draw down speed-trials

The following Table 6 presents the titer values, along with the breaking strength and elongation properties of the spun yarns within the draw-down speed trials. Titer, expressed in dtex, is a fundamental parameter describing the linear density of the yarn, which influences both its mechanical performance and end-use applications. The tenacity, defined as the maximum load per unit of titer, provides insight into the load-bearing capacity of the yarns before failure, which is crucial for assessing their durability in textile applications. Elongation at break reflects the ability of the yarns to stretch before failure, serving as an indicator of their flexibility and resistance to mechanical deformation.

**Table 6:** Mechanical properties of the yarns spun within the draw down speed-trials.

Trial-ID	Titer [dtex]	Titer CV [%]	Tenacity [cN/dtex]	Tenacity CV [%]	Elongation [%]	Elongation CV [%]
32.0999-1	537.7	0.16	0.891	6.03	363.2	4.06
32.0999-2	403.7	0.25	1.1	2.29	329.2	3.93
32.0999-3	323.5	0.58	1.241	3.08	303.2	3.38
32.0999-4	269.2	0.42	1.415	2.65	283.3	4.30
32.0999-5	229.1	0.39	1.422	3.30	256.7	5.93
32.0999-6	199.3	1.38	1.481	3.56	241.3	5.53
32.9002-1	539.8	0.25	0.772	3.48	379.2	5.21
32.9002-2	405.4	0.55	0.910	2.53	345.8	3.84
32.9002-3	268.4	0.81	1.108	3.47	301.6	6.39
32.9002-4	201.8	1.55	1.207	4.08	269.6	8.06
32.9002-5	159.6	1.98	1.246	2.36	234.2	7.07

**Fig. 6:**
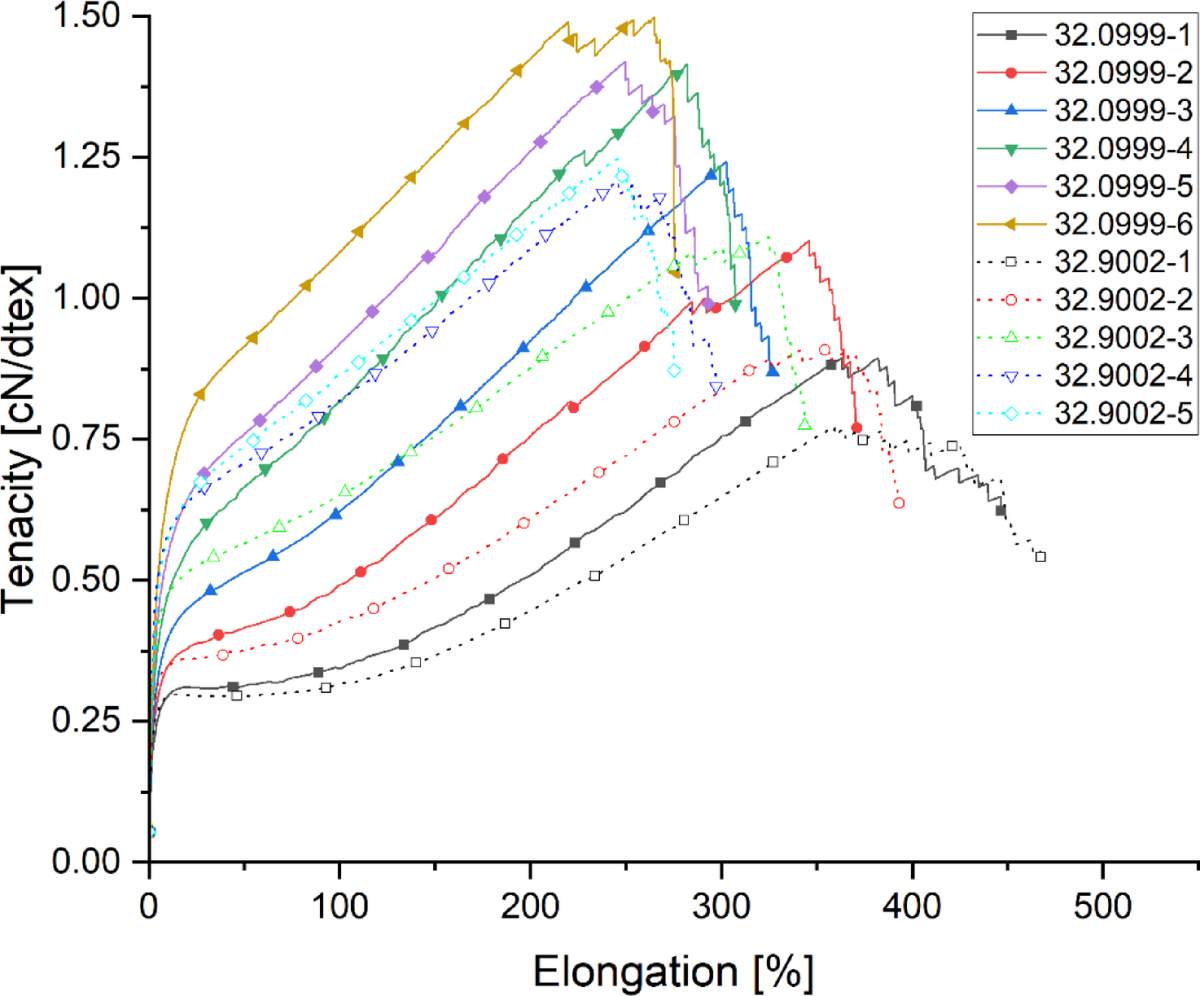
Tensile diagram of the yarns spun within the draw down speed-trials.

The tensile diagram (Fig. 6) provides a detailed understanding of the performance characteristics of the spun yarns, revealing a strong correlation between draw-down speed and yarn strength.

As predicted by polymer processing models, the increase in draw-off speed results in higher polymer chain alignment, leading to greater molecular orientation and enhanced tenacity. Specifically, increasing the draw-off speed v by a factor of 2.67 (from 750 m/min to 2000 m/min) leads to a 166% increase in tenacity. Simultaneously, a notable reduction in yarn titer is observed, indicating that the filaments become finer as they are stretched further during the spinning process. This is consistent with the theoretical expectation that increased extensional flow results in fiber thinning while improving tensile properties. However, a trade-off is evident in elongation at break, which decreases by 120%, demonstrating that the yarns become less ductile and more brittle as their molecular orientation increases. Furthermore, the results show that the strength of yarns spun from material 32.0999 is up to 20% higher than that of yarns spun from material 32.9002. Even at an increased draw-down speed of 2500 m/min, the yarns from material 32.9002 still exhibit lower tenacity and elongation at break. These findings highlight the importance of both material selection and process optimization in achieving high-performance rPP multifilament yarns suitable for industrial applications.

#### Mechanical properties of the yarns spun within the draw ratio-trials

The following Table 7 the titer values, along with the tenacity and elongation properties of the spun yarns within the draw ratio-trials.

**Table 7:** Mechanical properties of the yarns spun within the draw ratio-trials.

Trial-ID	Titer [dtex]	Titer CV [%]	Tenacity [cN/dtex]	Tenacity CV [%]	Elongation [%]	Elongation CV [%]
32.0999-7	282.2	0.41	2.018	4.86	136.6	11.8
32.0999-8	212.1	2.17	2.631	3.11	73	14.8
32.0999-9	175.4	1.02	3.336	1.95	33.3	12.68
32.0999-10	145.4	0.94	4.277	3.9	24.3	9.19

**Fig. 7:**
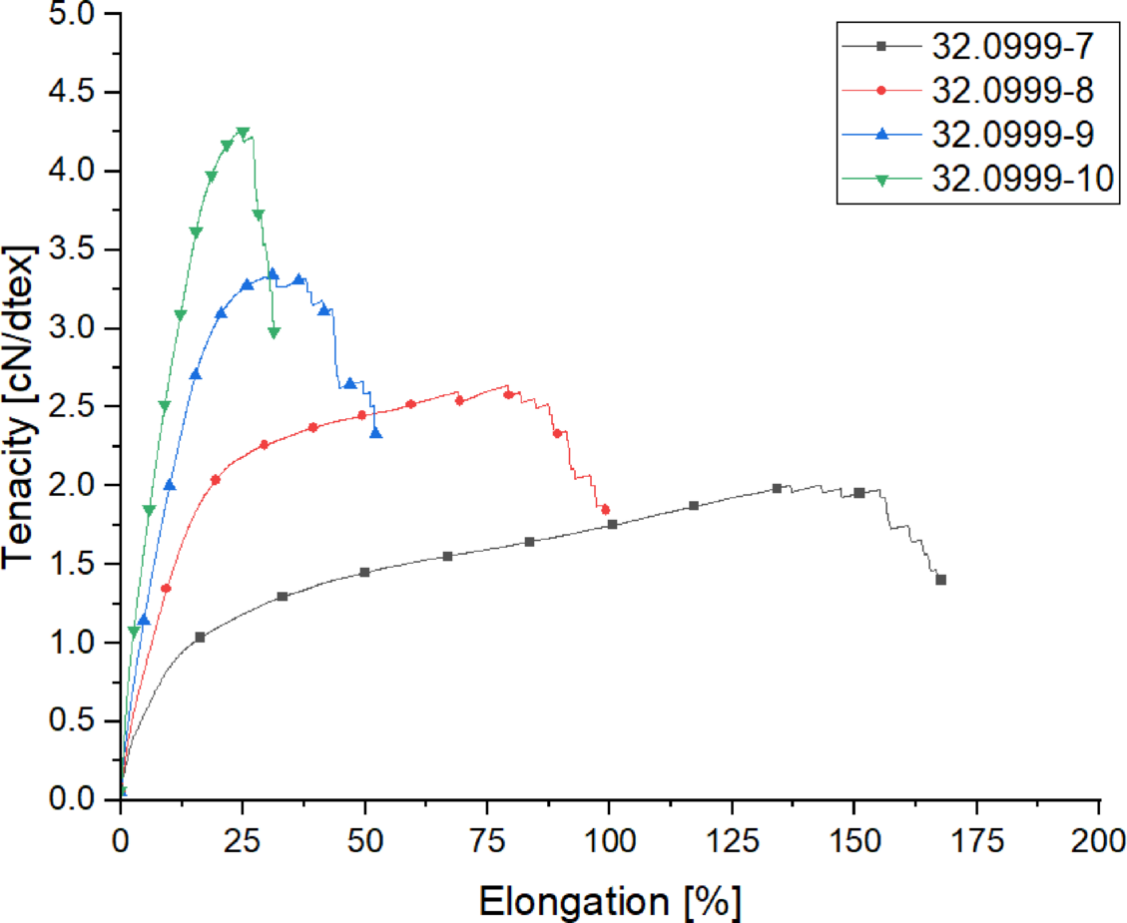
Tensile diagram of the yarns spun within the draw ratio-trials.

The draw ratio has a significant impact on the mechanical properties of spun multifilament yarns, as it determines the degree of molecular orientation and crystallinity within the filaments. Higher draw ratios lead to increased polymer chain alignment along the fiber axis, resulting in enhanced tenacity and stiffness while reducing elongation at break. This increased orientation improves the tensile strength of the yarns, making them more suitable for demanding textile applications. In this study (Fig. 7), a maximum tenacity of 4.2 cN/dtex was achieved, with a corresponding residual elongation of 24%. The analysis reveals a nearly linear relationship between DR4 and Tenacity, indicating that an increase in the draw ratio leads to a proportional increase in tenacity. Optimizing the draw ratio is essential to balancing filament strength and flexibility. A higher draw ratio enhances structural integrity by minimizing defects and promoting uniform stress distribution within the filaments. However, excessive stretching can induce brittleness, potentially limiting the yarn’s ability to withstand dynamic loading in textile applications. The results indicate that within the tested range, increasing the draw ratio effectively improves mechanical performance. This combination of high strength and moderate elongation ensures that the yarns can be efficiently processed while maintaining the necessary performance characteristics for high-quality textile production. Although higher draw ratios were not explored due to machine limitations, the results suggest that further increases may be feasible with appropriate process modifications.

#### Mechanical properties of the yarns spun within the draw temperature-trials

The following Table 8 presents the titer values, along with the breaking strength and elongation properties of the spun yarns within the draw temperature-trials.

**Table 8:** Mechanical properties of the yarns spun within the draw temperature-trials.

Trial-ID	Titer [dtex]	Titer CV [%]	Tenacity [cN/dtex]	Tenacity CV [%]	Elongation [%]	Elongation CV [%]
32.0999-11	178.6	0.40	3.180	2.84	62.6	7.08
32.0999-12	177.4	0.34	3.115	3.55	56.0	10.23
32.0999-13	171.3	0.52	3.684	1.71	38.1	12.79

The results clearly show the impact of the drawing temperature on both tenacity and elongation at break. While increasing the drawing temperature T_3_ from 60 to 90 °C shows no significant effect, a further increase to 130 °C exhibits a substantial influence on mechanical properties. At this higher temperature, the tenacity improves by approximately 20%, indicating a notable enhancement in yarn strength. This increase can be attributed to greater molecular orientation and crystallinity, which allows the yarns to sustain higher loads before breaking. According to polymer physics^33,34^, higher drawing temperatures facilitate the mobility of polymer chains, enabling their rearrangement into a more ordered crystalline structure. This results in stronger intermolecular forces and improved fiber strength. Similar findings have been reported in studies on melt-spun polyester and polyamide fibers, where an increase in drawing temperature correlates with enhanced tensile properties.

**Fig. 8:**
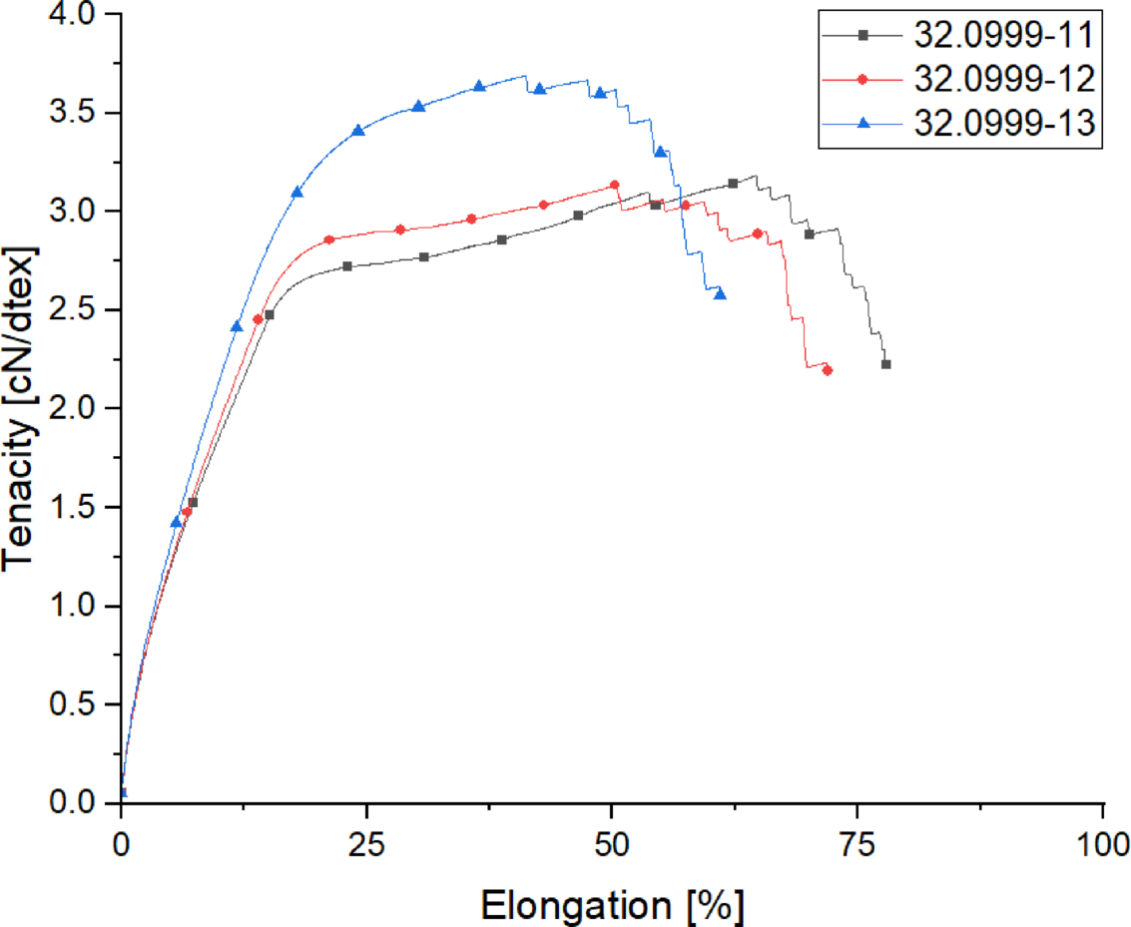
Tensile diagram of the yarns spun within the draw temperature-trials.

Conversely, the elongation at break is reduced by about 20% when the drawing temperature is elevated to 130 °C (Fig. 8). This decrease in elongation suggests that the yarns become less ductile and more brittle as the temperature increases. The higher temperature promotes a rigid and aligned molecular structure, which restricts chain mobility and reduces the yarn’s ability to stretch before failure. This effect is widely observed in thermoplastic fibers, where an increase in crystallinity leads to higher strength but lower deformability.

#### Mechanical properties of the yarns spun within the godet-trials

The following Table 9 presents the titer values, along with the breaking strength and elongation properties of the spun yarns within the godet-trials.

**Table 9:** Mechanical properties of the yarns spun within the godet-trials.

Trial-ID	Titer [dtex]	Titer CV [%]	Tenacity [cN/dtex]	Tenacity CV [%]	Elongation [%]	Elongation CV [%]
32.0999-14	282.2	0.41	2.018	4.86	136.6	11.80
32.0999-15	279.2	0.42	1.936	2.80	132.6	7.96
32.0999-16	284.6	0.83	1.746	3.59	129.9	11.35
32.0999-17	211.4	0.33	2.511	3.66	74.9	13.13
32.0999-18	195.1	1.14	2.578	3.56	49.0	20.20
32.0999-19	173.8	2.97	3.079	3.82	33.6	15.61

**Fig. 9:**
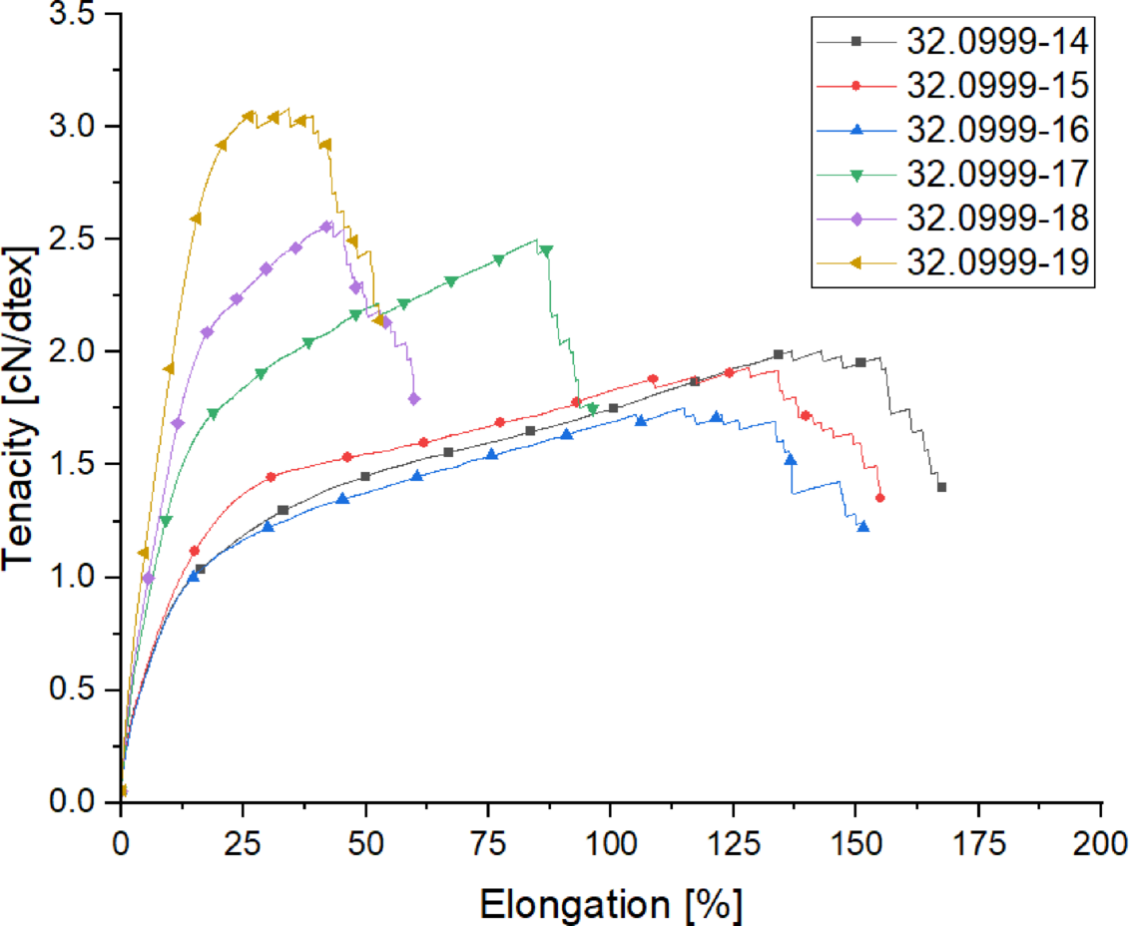
Tensile diagram of the yarns spun within the godet-trials.

The test series (Fig. 9) reveals that the sequence in which the drawing steps are performed, specifically in samples 32.0999-14 and 32.0999-15, does not have a significant impact on the mechanical properties of the yarns. This suggests that the order of the drawing stages within these conditions is not a critical factor influencing the outcome of the yarn’s performance. However, the results clearly indicate that a two-stage stretching process offers distinct advantages over a single-stage process.

For instance, sample 32.0999-16, which underwent a different drawing sequence, exhibits a 17% reduction in tenacity compared to yarns 32.0999-14 and 32.0999-15. This decrease in tenacity indicates that the yarn produced through a single-stage drawing process is weaker in terms of strength. The trend continues across the other tested yarns, with all samples subjected to a single-stage drawing process showing at least a 10% reduction in tenacity compared to their two-stage counterparts. These findings underscore the importance of the two-stage drawing process, which appears to enhance the molecular orientation and crystallinity of the yarns, leading to a better tenacity. The two-stage process allows for more controlled deformation, enabling the yarns to develop higher tenacity while maintaining structural integrity.

#### Mechanical properties of the yarns spun within the quenching-trials

**Fig. 10:**
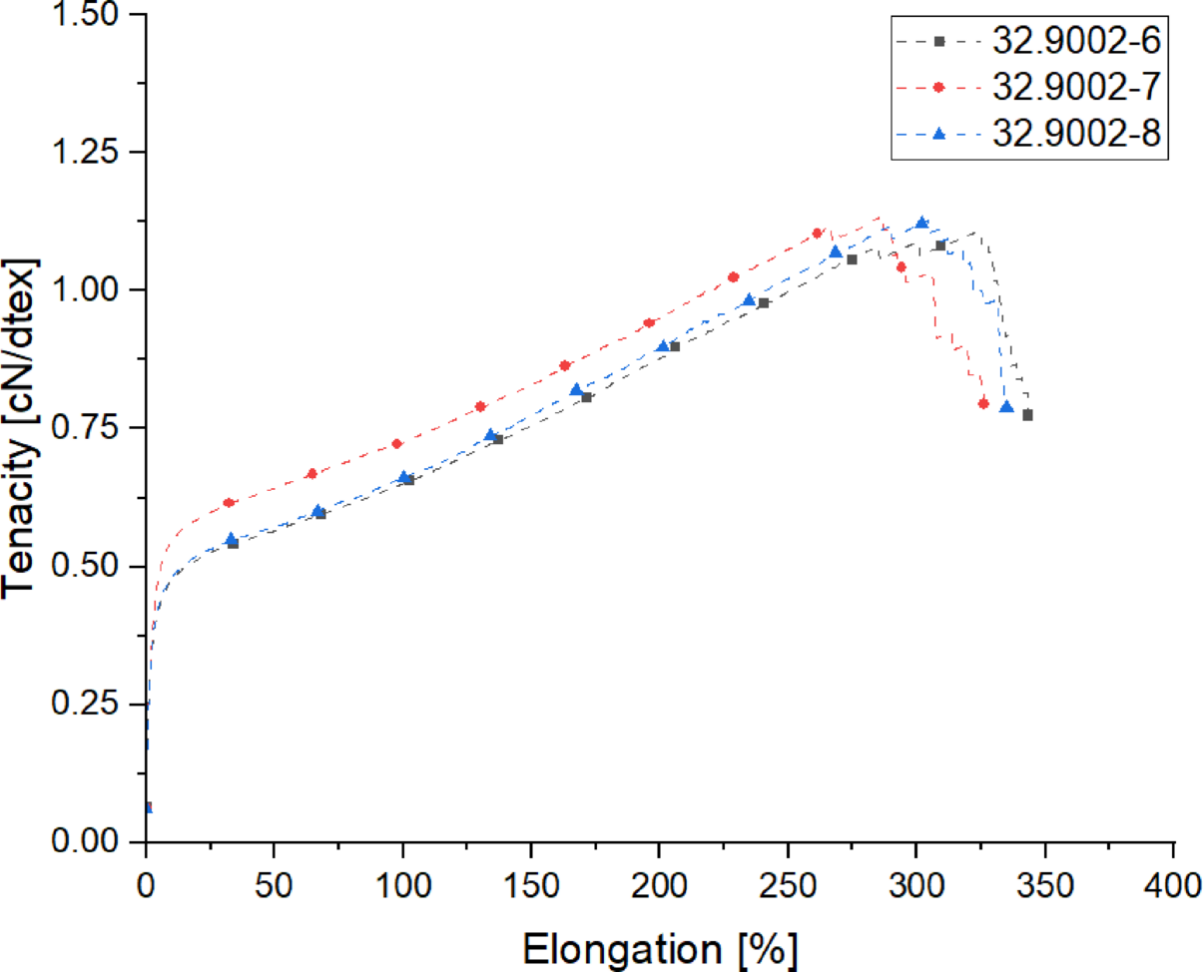
Tensile diagram of the yarns spun within the quenching-trials.

The following results (Table 10; Fig. 10) presents the titer values, along with the breaking strength and elongation properties of the spun yarns within the quenching-trials.

**Table 10:** Mechanical properties of the yarns spun within the quenching-trials.

Trial-ID	Titer [dtex]	Titer CV [%]	Tenacity [cN/dtex]	Tenacity CV [%]	Elongation [%]	Elongation CV [%]
32.9002-6	268.4	0.81	1.108	3.47	301.6	6.39
32.9002-7	264.0	0.53	1.137	2.35	292.0	5.16
32.9002-8	262.1	1.28	1.127	3.43	293.9	5.21

To validate the reliability of the measured mechanical properties, a statistical analysis was conducted using t-tests for pairwise comparisons between the tested quenching conditions. The results indicate that variations in quenching air did not lead to substantial differences in the mechanical properties of the yarns. For tenacity, a statistically significant difference (*p* = 0.0085) was observed between 32.9002 and 6 and 32.9002-7, while other comparisons showed no significant variations. In contrast, for elongation at break (%), no significant differences were detected across all tested conditions (*p* > 0.05), confirming that quenching air variations had no measurable impact on fiber ductility.

One potential explanation is the relatively low take-up speed (1500 m/min) used in these trials. At lower spinning speeds, the residence time in the quenching zone is inherently longer, allowing for sufficient cooling even at varying airflow rates. In contrast, at higher spinning speeds, quenching conditions can become more critical, as limited cooling time may impact the final crystallinity and molecular orientation.

## Conclusions

The results of this study demonstrate the successful processing of mechanically recycled polypropylene (rPP) from post-consumer waste into high-performance multifilament yarns without the need for compatibilizers or processing aids. The achieved tenacity of up to 4.2 cN/dtex aligns well with the typical range (1.5-6 cN/dtex) reported for textile-grade PP multifilament yarns, demonstrating the viability of mechanically recycled rPP for high-performance textile applications^29^. This finding provides new insights into the optimization of melt spinning parameters for industrial textile applications. This advancement opens new possibilities for integrating rPP into textile sectors where sustainability and resource efficiency are crucial, such as functional sportswear, automotive textiles, and geotextiles. The optimal processing parameters identified in this study can serve as a guideline for industrial melt spinning processes, contributing to the advancement of a circular economy in polypropylene-based textiles. The study’s key findings indicate that higher draw down speeds and increased drawing temperatures enhance tenacity but reduce elongation, highlighting a trade-off between strength and flexibility. Additionally, a two-stage drawing process improves mechanical properties, while variations in quenching air show minimal impact. Building on these results, future research should explore higher spinning speeds to improve process efficiency while maintaining mechanical performance. Another important aspect is the influence of rPP composition on spin dyeing, which is the standard coloring method for polypropylene fibres. Investigating how pigments interact with recycled polymer matrices could optimize color uniformity and stability, further enhancing the applicability of rPP in textile production. Additionally, the functionalization of fibres with conductive fillers such as carbon nanotubes (CNTs) could enable the production of electrically conductive yarns, opening up new possibilities for smart textiles and sensor applications. Future research could also explore the use of rPP multifilament yarns as reinforcement in bio-based composite materials, similar to approaches demonstrated in studies on laminated composite sandwich plates^35,36^. By addressing these aspects, future research can contribute to an even broader utilisation of rPP yarns in advanced textile applications, reinforcing the role of recycled polymers in sustainable and high-performance materials.

## Data Availability

The dataset is available from the corresponding author on reasonable request.
